# Association of Hearing Loss and Tinnitus with Health-Related Quality of Life: The Korea National Health and Nutrition Examination Survey

**DOI:** 10.1371/journal.pone.0131247

**Published:** 2015-06-29

**Authors:** Young-Hoon Joo, Kyung-do Han, Kyung Ho Park

**Affiliations:** 1 Department of Otolaryngology-Head and Neck Surgery, College of Medicine, The Catholic University of Korea, Seoul, Korea; 2 Department of Biostatistics, College of Medicine, The Catholic University of Korea, Seoul, Korea; University of Salamanca- Institute for Neuroscience of Castille and Leon and Medical School, SPAIN

## Abstract

**Background:**

Hearing loss and tinnitus are global public health concerns. There have been some studies suggesting a relationship between hearing loss and tinnitus and impaired health-related quality of life (HRQoL), but there have been no large cross-sectional epidemiologic studies of a representative sample of the entire country population investigating this possible association.

**Objective:**

The aim of this study was to investigate the relationship between hearing loss and tinnitus and HRQoL in South Korea using data from the Korea National Health and Nutrition Examination Surveys during 2010–2012.

**Methods:**

Cross-sectional data of 11,266 adults who completed the Korea National Health and Nutrition Examination Surveys were analyzed. Subjects were divided into four groups as follows: normal hearing without tinnitus, normal hearing with tinnitus, hearing loss without tinnitus, and hearing loss with tinnitus.

**Results:**

Among the population that was ≥19 years of age, the prevalence of unilateral hearing loss was 9.69% and that of tinnitus in the prior 12 months was 32.76%. The hearing loss with tinnitus group had the highest percentage of subjects who responded “some or extreme problems” in all five dimensions (mobility, self-care, usual activities, pain/discomfort, and anxiety/depression) of HRQoL. After adjustment for age, gender, body mass index, smoking status, alcohol intake, regular exercise, house income, education level, diabetes, hypertension, and stress level, the HRQoL odds ratios (OR) were 1.47 (95% confidence interval [CI], 1.07–2.02) for mobility, 1.59 (95% CI, 1.07–2.37) for usual activity, and 1.84 (95% CI, 1.25–2.70) for anxiety/depression in the hearing loss with tinnitus group, compared with the normal hearing without tinnitus group. The ORs for the normal hearing with tinnitus group compared with the hearing loss without tinnitus group was increased in all five dimensions of HRQoL after adjustment for confounders.

**Conclusion:**

Hearing loss with tinnitus has a considerable impact on HRQoL in the Korean population. In our study, the hearing loss without tinnitus group showed better a HRQoL than the normal hearing with tinnitus group.

## Introduction

Hearing loss and tinnitus continue to be bothersome and challenging clinical problems. Hearing loss can interfere with the ability to understand speech sounds, leading to difficulties in communication and learning, reduced work productivity, increased depression and anxiety, and social isolation [1]. Age-related hearing loss is one of the three leading common chronic diseases in elderly individuals, along with arthritis and hypertension, and its incidence is increasing rapidly [2]. More than 35% of people in their 60s and 50% of those in their 70s have difficulties in daily activities resulting from hearing loss [2]. Tinnitus, the perception of sound from the ears or head without an audible external source, is a symptom, rather than a disease entity, that originates internally without external auditory input. Tinnitus affects an estimated 12–15% of the general population in the United States and Europe and more than a third of the population older than age 65 [3,4]. Since 1948, when the World Health Organization defined health as being not only the absence of disease and infirmity but also the presence of physical, mental, and social well-being, health-related quality of life (HRQoL) issues have become steadily more important in health care practice [5]. Impaired HRQoL has been associated with increased mortality and disease progression [6,7].

The Korean National Health and Nutrition Examination Surveys (KNHANES), which is a government-driven survey by the Korea Center for Disease Control and Prevention, has been conducted since 1998. The Korean Society of Otorhinolaryngology—Head and Neck Surgery participated in this project, and interviews and examinations were performed by trained ear, nose, and throat residents. KNHANES (2010) was the first population-based study to use a questionnaire for determining hearing loss and tinnitus as well as HRQoL such as people’s level of mobility, self-care, usual activities, pain/discomfort, and anxiety/depression in the noninstitutionalized civilian population of South Korea. Although people with hearing loss and tinnitus are at an increased risk of experiencing HRQoL problems, population-based studies for hearing loss and tinnitus are lacking. The aim of this study was to analyze the prevalence of hearing loss and tinnitus and its potential risk factors, including lifestyle habits and anthropometric measurements, using KNHANES data. In addition, the association of hearing loss with HRQoL was also evaluated based on the questionnaire in South Korea.

## Materials and Methods

### Ethics Statement

The study protocol was approved by the Institutional Review Board of the Catholic Medical Center.

### Study Population

This study was based on data collected during the 2010–2012 KNHANES. Conducted by the Division of Chronic Disease Surveillance under the Korea Centers for Disease Control and Prevention since 1998, the KNHANES is a nationwide survey designed to accurately assess national health and nutrition levels. A field survey team that included an otolaryngologist, an ophthalmologist, and nurse examiners for health assessments moved with a mobile examination unit and performed interviews and physical examinations. The survey consists of a health interview, a nutritional survey, and a health examination survey. The survey amasses data via household interviews and by direct standardized physical examinations conducted in specially equipped mobile examination centers. The KNHANES methodology has been described in detail previously [8–10].

The sample included 19,599 participants older than 19 years. Among these selected individuals, analyses were conducted of the data from 11,266 participants who agreed to the clinical examination and answered the questionnaire on HRQoL and presence of tinnitus. Written informed consent was obtained from all participants before the survey, and approval for this study was obtained from the Institutional Review Board of the Catholic University of Korea in Bucheon, Korea.

### Survey for Tinnitus

Participants were asked about their tinnitus experiences. In response to the questionnaire item, “Within the past year, did you ever hear a sound (buzzing, hissing, ringing, humming, roaring, machinery noise) originating in your ear?,” examiners were instructed to record “yes” if a participant reported hearing an odd or unusual noise at any time in the past year. Participants who responded positively to this question were then queried concerning the resulting annoyance in their lives by the following questions: “How severe is this noise in daily life?” (not annoying/annoying [irritating]/severely annoying and causes sleep problem). The participants were grouped as having annoying tinnitus if the severity of tinnitus was annoying or severely annoying.

### Audiometric Measurement

Pure-tone audiometric testing was conducted using an SA 203 audiometer (Entomed; Malmö, Sweden). Testing was conducted in a soundproof booth inside the mobile unit reserved for the KNHANES. Otolaryngologists, who had been trained to operate the audiometer, provided instructions to participants and obtained the recordings. All audiometric testing was performed under the supervision of an otolaryngologist. Only air conduction thresholds were measured. Supra-auricular headphones were used in the soundproof booth. The otolaryngologist provided basic instructions to the participant regarding the automated hearing test. Automated testing was programmed according to a modified Hughson-Westlake procedure; it used a single, pure tone of 1–2 seconds. The lowest level at which the subject responded to 50% of the pure tone was set as the threshold. The automated hearing test involving air-conducted pure-tone stimuli showed good test—retest reliability and validity comparable to the manual pure-tone audio test [11,12]. Participants responded by pushing a button when they heard a tone and the results were automatically recorded. The frequency ranges tested were 0.5, 1, 2, 3, 4, and 6 kHz. We defined hearing loss as the pure-tone averages of frequencies at 0.5, 1, 2, and 4 kHz at a threshold of a ≥40 decibel hearing level in the ear with worse hearing.

### HRQoL Survey

HRQoL was evaluated using the EuroQol, which consists of two parts: the health-status descriptive system (EuroQoL 5-dimension, EQ-5D) and the EQ visual analog scale. The EQ-5D records the level of self-reported problems according to five dimensions (mobility, self-care, usual activities, pain/discomfort, and anxiety/depression) [13,14]. Each dimension is assessed based on a single question with three response levels (no problem, some problems, and extreme problems). Additionally, the EQ-5D questionnaire contains a visual analog scale ranging from 0 (worst imaginable health state) to 100 (best imaginable health state), which enables respondents to assess their health subjectively.

### Lifestyle Habits

Medical history and lifestyle habits information were collected using self-reported questionnaires. Smoking history was categorized into three groups: current smoker, ex-smoker, or nonsmoker. Based on the amount of alcohol consumed per day during the 1-month period before the interview, the subjects were classified into three groups: nondrinkers, mild to moderate drinkers (<15 g/day), or heavy drinkers (≥15 g/day). Regular exercise was defined as strenuous physical activity performed for at least 20 minutes at a time at least three times a week. Weight and height were measured by well-trained medical professionals. Standing height was measured when the subject faced directly ahead with shoes off, feet together, arms by the sides, and heels, buttocks, and upper back in contact with the wall. The unit of height was measured in centimeters with one decimal point using the SECA 225 (SECA, Hamburg, Germany). Body mass index was calculated as weight (kg)/height (m^2^).

### Statistical analysis

Statistical analyses were performed using the SAS survey procedure (version 9.3; SAS Institute, Cary, NC) to reflect the complex sampling design and sampling weights of the KNHANES and provide nationally representative prevalence estimates. The procedures included unequal probabilities of selection, oversampling, and nonresponse so that inferences could be made about the Korean adolescent participants.

The prevalence and 95% confidence intervals (CIs) for tinnitus were calculated. In the univariate analysis, the Rao-Scott chi-square test (using PROC SURVEYFREQ in SAS) and logistic regression analysis (using PROC SURVEYLOGISTIC in SAS) were used to test the association between hearing, tinnitus, and HRQoL in a complex sampling design. Participants’ characteristics were described using means and standard errors for continuous variables and numbers and percentages for categorical variables. We first adjusted for age and gender (model 1) and then adjusted for the variables in model 1 plus smoking status, alcohol intake, physical activity, body mass index (model 2), and then for model 2 plus income, education level diabetes, hypertension, and stress level (model 3).

## Results

### General Characteristics of the Study Population

Among the 11,266 participants ≥19 years of age, the prevalence of unilateral hearing loss was 9.69% and that of tinnitus in the prior 12 months was 32.76%. In both the presence of tinnitus and the absence of tinnitus groups, subjects with hearing loss were older and less likely to exercise regularly, more likely to have a low income, and more likely to have chronic disease such as diabetes or hypertension compared with subjects with normal hearing (Table 1). In both the normal hearing and hearing loss groups, subjects with tinnitus had a higher percentage of moderate to severe stress, depressive mood, suicide ideation, episodes of falling, and history of dizziness than those without tinnitus.

**Table 1:** Analysis of factors potentially associated with hearing and tinnitus.

	Normal hearing tinnitus (-)	Normal hearing tinnitus (+)	Hearing loss tinnitus (-)	Hearing loss tinnitus (+)	
	n = 7917	n = 2354	n = 569	n = 426	P value
Age (years)	54.0 ± 0.2	57.1 ± 0.3	67.0 ± 0.7	66.8 ± 0.9	<0.0001*
Gender: male (%)	49.0 ± 0.6	45.5 ± 1.2	53.2 ± 2.9	52.3 ± 3.0	0.0156*
Smoking: current smoker (%)	20.9 ± 0.6	19.9 ± 1.2	22.0 ± 2.6	19.7 ± 2.9	0.8288
Drinking: heavy drinker (%)	9.8 ± 0.4	8.2 ± 0.9	9.0 ± 1.9	8.1 ± 1.9	0.3688
Routine exercise (%)	19.8 ± 0.6	18.7 ± 1.1	15.5 ± 1.9	13.3 ± 2.0	0.0168*
Body mass index ≥25 kg/m^2^ (%)	36.3 ± 0.7	35.0 ± 1.2	32.9 ± 2.5	33.4 ± 3.1	0.4164
Diabetes (%)	11.9 ± 0.5	13.6 ± 1.0	18.5 ± 2.0	20.8 ± 3.0	<0.0001*
Hypertension (%)	37.4 ± 0.8	41.0 ± 1.3	57.5 ± 2.7	54.7 ± 3.1	<0.0001*
Education ≤middle school (%)	41.2 ± 1.0	52.1 ± 1.5	75.6 ± 2.6	72.7 ± 2.9	<0.0001*
Residential area: urban (%)	77.3 ± 1.9	74.8 ± 2.4	67.8 ± 3.3	71.3 ± 3.6	0.0003*
Income: lower quartile (%)	16.9 ± 0.7	26.4 ± 1.2	39.7 ± 2.5	43.3 ± 3.1	<0.0001*
Marital status with spouse (%)	86.4 ± 0.5	82.1 ± 1.0	69.6 ± 2.5	71.0 ± 2.7	<0.0001*
Stress: moderate to severe (%)	22.8 ± 0.6	28.9 ± 1.1	17.3 ± 2.1	30.9 ± 3.0	<0.0001*
Depressive mood (%)	12.7 ± 0.4	19.3 ± 1.0	13.1 ± 1.7	20.9 ± 2.4	<0.0001*
Suicide ideation (%)	12.9 ± 0.5	19.6 ± 1.1	20.3 ± 2.3	23.0 ± 2.7	<0.0001*
Falling attack (%)	1.7 ± 0.2	4.5 ± 0.6	2.0 ± 0.6	4.8 ± 1.3	<0.0001*
History of dizziness (%)	10.3 ± 0.5	22.0 ± 1.2	16.0 ± 2.0	27.5 ± 2.8	<0.0001*
EuroQoL 5-dimension index	74.3 ± 0.3	70.3 ± 0.5	67.3 ± 1.4	67.1 ± 1.2	<0.0001*
EuroQoL visual analog scale	0.94 ± 0.0	0.91 ± 0.0	0.88 ± 0.0	0.86 ± 0.0	<0.0001*

Data are presented as mean ± standard error.

* Significant at p<0.05.

### Prevalence of EQ-5D According to the Hearing and Tinnitus Subgroups

**Fig 1:**
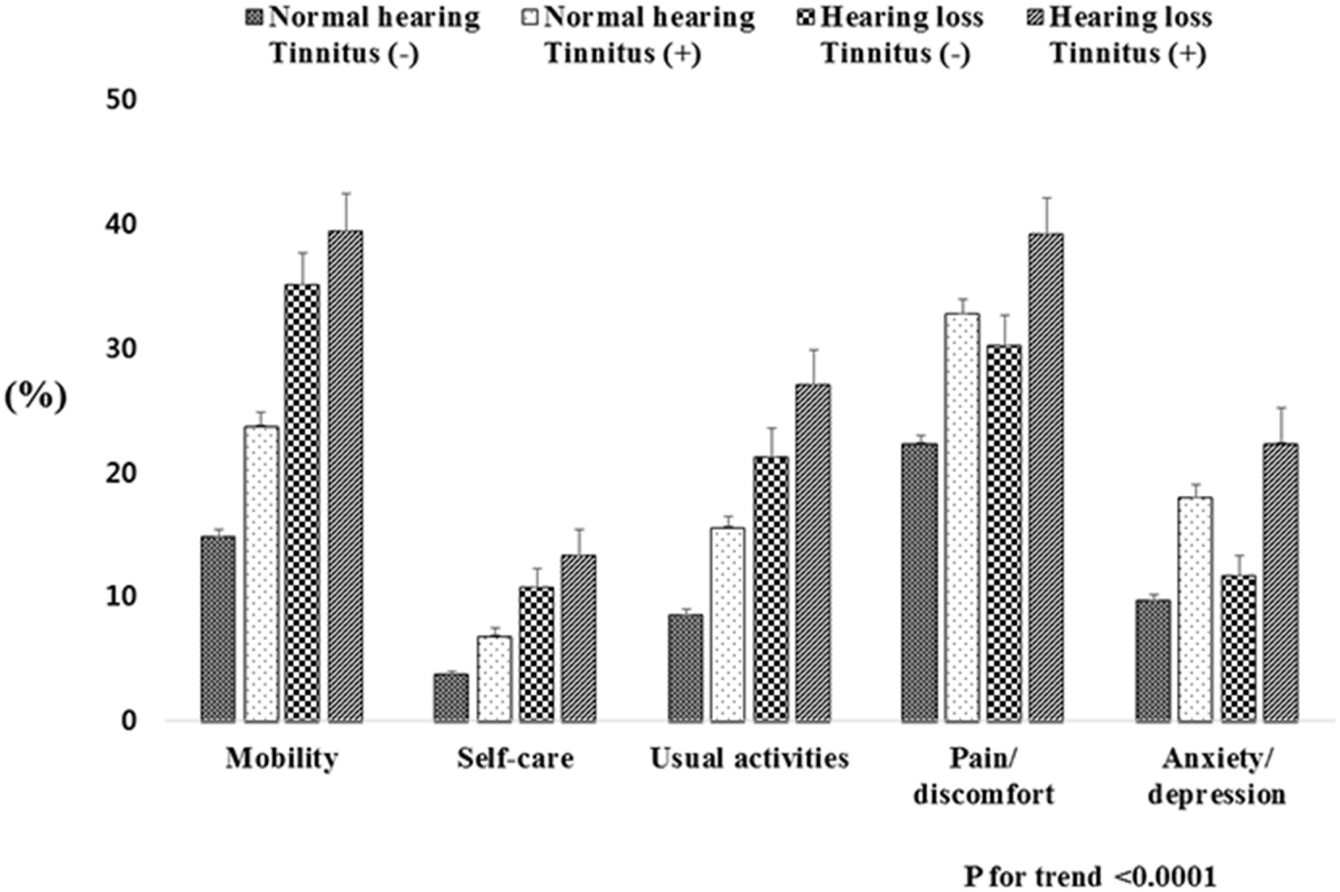
Prevalence of health-related quality of life according to four categories based on hearing status and presence of tinnitus.

Subjects were divided into four groups according to the hearing status and the presence of tinnitus: normal hearing without tinnitus, normal hearing with tinnitus, hearing loss without tinnitus, and hearing loss with tinnitus. The hearing loss with tinnitus group had the highest percentage of subjects who responded “some or extreme problems” in all five dimensions of EQ-5D. In the hearing loss with tinnitus group, the overall EQ-5D levels were as follows: 39.4% for mobility, 13.3% for self-care, 27.1% for usual activities, 39.2% for pain/discomfort, and 22.4% for anxiety/depression (Fig 1). The hearing loss with tinnitus group showed a higher proportion of “some or extreme problems” in the dimensions of anxiety/depression of the EQ-5D than the hearing loss without tinnitus group (p = 0.002).

**Fig 2:**
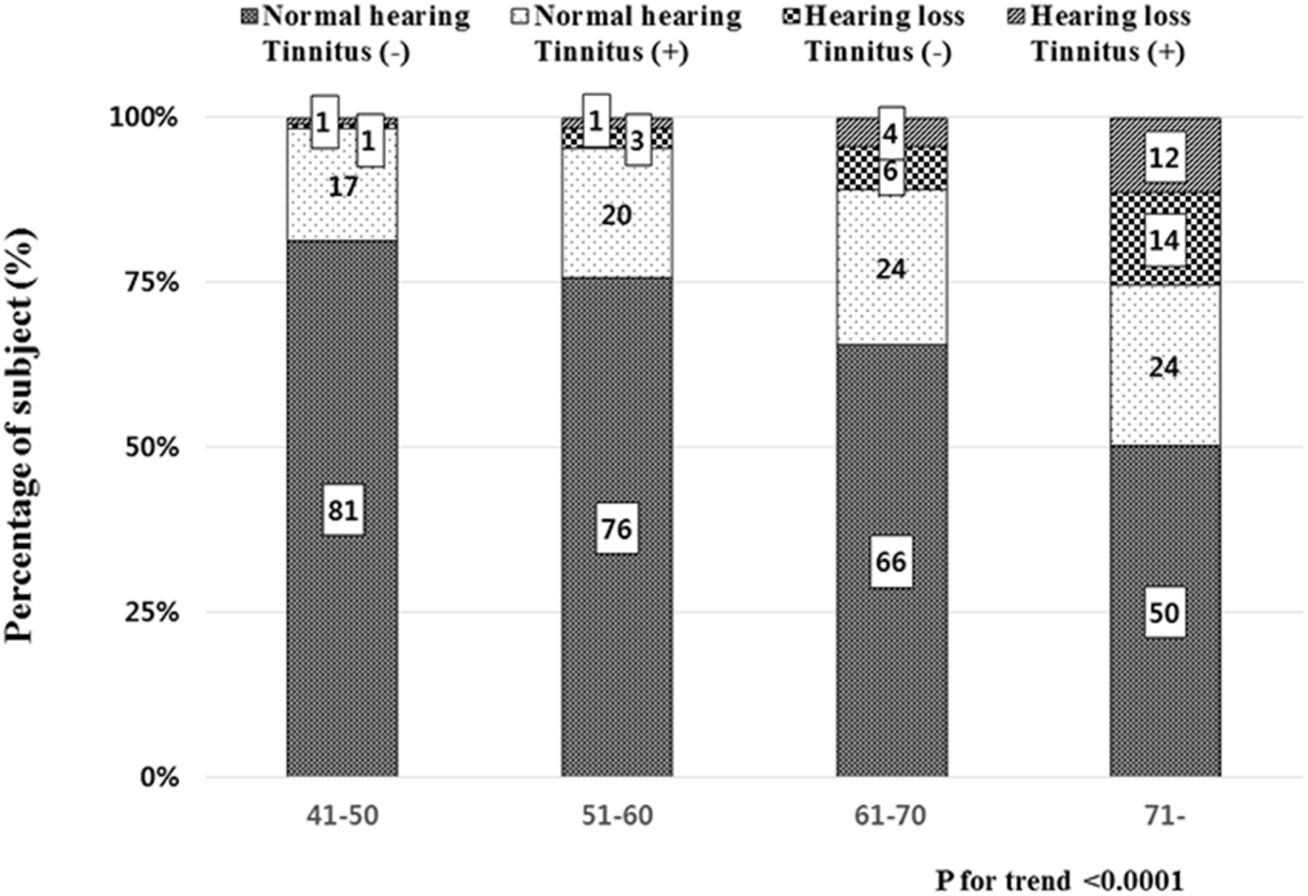
The distribution of the four categories according to age groups.

We analyzed the distribution of those four categories according to age groups. The proportion of subjects with normal hearing without tinnitus decreased significantly from 81% in the younger age group (41–50 years) to 50% in the older age group (≥71 years) (P for trend <0.0001; Fig 2). The proportion of subjects with normal hearing with tinnitus, hearing loss without tinnitus, and hearing loss with tinnitus groups increased linearly from the younger aged group to the older aged group.

### Multivariable Analyses for the Associations between EQ-5D and Hearing and Tinnitus Subgroups

Table 2 presents the differences in EQ-5D according to hearing and tinnitus after adjustment for confounders. After adjustment for age, gender, body mass index, smoking status, alcohol intake, regular exercise, house income, education level, diabetes, hypertension, and stress level, the OR values of the EQ-5D items were 1.47 (95% CI, 1.07–2.02) for mobility, 1.59 (95% CI, 1.07–2.37) for usual activity, and 1.84 (95% CI, 1.25–2.70) for anxiety/depression in the hearing loss with tinnitus group compared with the normal hearing without tinnitus group. The ORs for normal hearing with tinnitus group compared with the normal hearing without tinnitus group was also increased in all five dimensions of the EQ-5D after adjustment for confounders. However, the ORs for hearing loss without tinnitus group were not significantly increased in all five dimensions of the EQ-5D.

**Table 2:** Adjusted odd ratios and 95% confidence intervals for health-related quality of life.

		Normal hearing tinnitus (-)	Normal hearing tinnitus (+)	Hearing loss tinnitus (-)	Hearing loss tinnitus (+)	P for trend
Model 1						
	Mobility	1	1.43 (1.24–1.65)	1.23 (0.95–1.58)	1.52 (1.13–2.04)	<0.0001
	Self-care	1	1.45 (1.14–1.85)	1.15 (0.79–1.69)	1.50 (1.00–2.26)	<0.0001
	Usual activity	1	1.59 (1.34–1.88)	1.18 (0.87–1.59)	1.69 (1.22–2.35)	<0.0001
	Pain/discomfort	1	1.50 (1.31–1.72)	0.92 (0.72–1.18)	1.41 (1.10–1.81)	<0.0001
	Anxiety/depression	1	1.87 (1.60–2.19)	0.93 (0.66–1.31)	2.05 (1.44–2.91)	<0.0001
Model 2						
	Mobility	1	1.43 (1.24–1.66)	1.20 (0.93–1.56)	1.59 (1.18–2.15)	<0.0001
	Self-care	1	1.43 (1.12–1.83)	1.15 (0.79–1.68)	1.52 (1.00–2.30)	<0.0001
	Usual activity	1	1.58 (1.33–1.87)	1.14 (0.84–1.55)	1.74 (1.24–2.43)	<0.0001
	Pain/discomfort	1	1.50 (1.31–1.71)	0.90 (0.70–1.15)	1.44 (1.12–1.86)	<0.0001
	Anxiety/depression	1	1.86 (1.59–2.18)	0.94 (0.67–1.32)	2.08 (1.46–2.96)	<0.0001
Model 3						
	Mobility	1	1.28 (1.09–1.49)	1.11 (0.84–1.48)	1.47(1.07–2.02)	<0.0001
	Self-care	1	1.32 (1.00–1.74)	1.16 (0.76–1.77)	1.38 (0.86–2.23)	<0.0001
	Usual activity	1	1.45 (1.21–1.75)	1.12 (0.79–1.59)	1.59 (1.07–2.37)	<0.0001
	Pain/discomfort	1	1.39 (1.21–1.60)	0.87 (0.66–1.15)	1.28 (0.96–1.71)	<0.0001
	Anxiety/depression	1	1.64 (1.39–1.93)	0.97 (0.65–1.43)	1.84 (1.25–2.70)	<0.0001

Model 1 is adjusted for age and sex.

Model 2 is adjusted for age, sex, body mass index, smoking status, alcohol intake, and regular exercise.

Model 3 is adjusted for age, sex, body mass index, smoking status, alcohol intake, regular exercise, house income, education level, diabetes, hypertension, and stress level.

## Discussion

This study of a population-based sample of adults in Korea aged 19 years or older found an association between hearing loss and tinnitus and disability with HRQoL. Our results from a large cross-sectional study of a representative sample of the Korean population support those from previous smaller trials that have demonstrated the association between hearing loss and tinnitus and HRQoL. HRQoL is used as an outcome measurement that encompasses physical, emotional, and social dimensions in different populations. In patient populations, it is used to assess the relative burden a chronic disease has on HRQoL or the effect of a new treatment [15].

Several studies have found associations between hearing loss or tinnitus with worse subjective and objective measures of physical and psychological HRQoL. Chia et al. reported an association between hearing impairment and decreased physical and mental health scores of HRQoL [16]. Sindhusake et al. showed that hearing impairment and vertigo increased the risk for severe tinnitus [17]. Carlsson et al. showed that annoying tinnitus, defined as affecting daily life often or always, and remaining vertigo were the strongest predictors of negative effects on HRQoL after sudden sensorineural hearing loss [18]. Tinnitus is frequently associated with hearing impairment and sometimes with vertigo. The extent to which tinnitus impairs HRQoL is highly variable. Many people remain unaffected by the phantom sounds, whereas others are severely impaired and may even become suicidal [19]. A study using the Short Form Health Survey for assessing patients’ HRQoL showed that 43% of patients with tinnitus also had impaired HRQoL or a high level of distress or both [20]. Furthermore, many tinnitus patients are known to suffer from insomnia, which has a considerable impact on the HRQoL [21,22]. In this cohort the hearing loss without tinnitus group had a higher proportion of subjects who responded “some or extreme problems” than the normal hearing with tinnitus group in the dimensions of mobility, self-care, and usual activities of the EQ-5D by univariate analysis. Subjects with hearing loss were older than those with normal hearing. The older age group generally had more comorbidities and other risk factors; therefore, these conditions might have caused the much poorer HRQoL in older subjects with tinnitus than younger subjects with tinnitus. However, by using multiple logistic regression analyses after adjusting for age, there was no significant association between the hearing loss without tinnitus and all five dimensions of HRQoL.

We demonstrated that HRQoL in the hearing loss with tinnitus group was significantly impaired compared with the normal hearing without tinnitus group after adjusting for sociodemographic factors and comorbidities. In the subgroup analyses, a significant deterioration of HRQoL was observed in the tinnitus group. When adjusted for age and gender in model 1, the normal hearing with tinnitus group had a greater OR in all five dimensions of HRQoL than the hearing loss without tinnitus group. Furthermore, there was no significant effect of hearing loss without tinnitus on all five dimensions of HRQoL after adjusting for sociodemographic factors and comorbidities. Our data indicate that HRQoL has a closer relationship with tinnitus than hearing loss. Many authors reported that hearing impairment increased burden of communication and poorer HRQoL [16–18]. This difference may have occurred because our data included participants with unilateral hearing loss compared to other studies. Most studies about the relationship between the hearing loss and HRQoL included participants with bilateral hearing loss.

This study has several limitations. First, we do not know the relative severity or grade of tinnitus in the absence of objective testing and more detailed questions. Second, the present study was cross-sectional. Although the causal relationship of risk factors with hearing loss or tinnitus is inconclusive, the results may be reliable because this is a population-based study from all parts of Korea. Third, there may be some response bias when reporting some parameters such as lifestyle habits and psychological stress because the KNHANES was conducted by using self-administered questionnaires.

In conclusion, subjects with hearing loss with tinnitus appear to have a greater risk of deterioration of HRQoL than those patients who simply have hearing loss or tinnitus. Tinnitus compared with hearing loss has a strong association with a psychological health of HRQoL.
